# Development of Dapagliflozin Solid Lipid Nanoparticles as a Novel Carrier for Oral Delivery: Statistical Design, Optimization, In-Vitro and In-Vivo Characterization, and Evaluation

**DOI:** 10.3390/ph15050568

**Published:** 2022-05-02

**Authors:** Aziz Unnisa, Ananda K. Chettupalli, Turki Al Hagbani, Mohammad Khalid, Suresh B. Jandrajupalli, Swarnalatha Chandolu, Talib Hussain

**Affiliations:** 1 Department of Pharmaceutical Chemistry, College of Pharmacy, University of Hail, Hail 81442, Saudi Arabia; 2 Department of Pharmaceutical Sciences, School of Pharmacy, Anurag University, Hyderabad 500088, India; anandphd88@gmail.com; 3 Department of Pharmaceutics, College of Pharmacy, University of Hail, Hail 81442, Saudi Arabia; t.alhagbani@uoh.edu.sa; 4 Department of Pharmacognosy, College of Pharmacy, Prince Sattam Bin Abdulaziz University, Al-Kharj 11942, Saudi Arabia; m.khalid@psau.edu.sa; 5 Department of Preventive Dental Sciences, College of Dentistry, University of Hail, Hail 81442, Saudi Arabia; s.jandrajupalli@uoh.edu.sa (S.B.J.); s.chandolu@uoh.edu.sa (S.C.); 6 Department of Pharmacology and Toxicology, College of Pharmacy, University of Hail, Hail 81442, Saudi Arabia; mdth_ah@yahoo.com

**Keywords:** dapagliflozin, solid lipid nanoparticles, Box–Behnken design, FTIR, DSC, XRD, SEM, AFM, in vitro Franz diffusion cells

## Abstract

Controlling hyperglycemia and avoiding glucose reabsorption are significant goals in type 2 diabetes treatments. Among the numerous modes of medication administration, the oral route is the most common. Introduction: Dapagliflozin is an oral hypoglycemic agent and a powerful, competitive, reversible, highly selective, and orally active human SGLT2 inhibitor. Dapagliflozin-loaded solid lipid nanoparticles (SLNs) are the focus of our present investigation. Controlled-release lipid nanocarriers were formulated by integrating them into lipid nanocarriers. The nanoparticle size and lipid utilized for formulation help to regulate the release of pharmaceuticals over some time. Dapagliflozin-loaded nanoparticles were formulated by hot homogenization followed by ultra-sonication. The morphology and physicochemical properties of dapagliflozin-SLNs have been characterized using various techniques. The optimized dapagliflozin-SLNs have a particle size ranging from 100.13 ± 7.2 to 399.08 ± 2.4 nm with 68.26 ± 0.2 to 94.46 ± 0.7% entrapment efficiency (%EE). Dapagliflozin-SLNs were optimized using a three-factor, three-level Box–Behnken design (BBD). Polymer concentration (X1), surfactant concentration (X2), and stirring duration (X3) were chosen as independent factors, whereas %EE, cumulative drug release (%CDR), and particle size were selected as dependent variables. Interactions between drug substances and polymers were studied using Fourier transform infrared spectroscopy (FTIR) and scanning electron microscopy (SEM). Differential scanning calorimetry (DSC), X-ray diffraction (XRD), and atomic force microscopy (AFM) analysis indicated the crystalline change from the drug to the amorphous crystal. Electron microscope studies revealed that the SLNs’ structure is nearly perfectly round. It is evident from the findings that dapagliflozin-SLNs could lower elevated blood glucose levels to normal in STZ-induced diabetic rats, demonstrating a better hypoglycemic impact on type 2 diabetic patients. The in vivo pharmacokinetic parameters of SLNs exhibited a significant rise in C_max_ (1258.37 ± 1.21 mcg/mL), AUC (5247.04 mcg/mL), and oral absorption (2-fold) of the drug compared to the marketed formulation in the Sprague Dawley rats.

## 1. Introduction

The Food and Drug Administration (FDA) approved dapagliflozin in 2014 as a novel oral hypoglycemic medication. In terms of structure, it is a tetrahydro-2H-pyran-3, 4, 5-triol fitting to the gliflozin family of compounds. Type 2 diabetes mellitus (T2DM) is currently treated with this drug. Dapagliflozin’s poor oral bioavailability is due to its poor solubility and stability [1]. Dapagliflozin increases urine glucose excretion and lowers blood glucose levels by blocking the transporter protein sodium-glucose co-transporter-2 (SGLT2) in the proximal renal tubule, inhibiting renal glucose reabsorption. Extensive follow-up periods of 1–4 years suggested that the dapagliflozin effects are sustained, and the drug is typically well-tolerated, making it a desirable therapy of choice.

Increased blood sugar levels (>70–110 mg/dL) are connected with the peril of micro-vascular impairment such as nephropathy, neuropathy, and retinopathy in T2DM [2,3]. About 95% of T2DM cases are found in the elderly, caused by genetics, fat, and lifestyle habits [4]. Anti-diabetic medications are currently used to treat diabetes, and their mechanisms include an increase in insulin production, lowering insulin resistance, and blocking glucose reabsorption from the Henley loop [5].

Researchers have observed various lipid-based nano preparations for improving the solubility, absorption, and dissolution characteristics of poorly soluble substances [6,7,8]. The SLNs have a nano size, a unique structure, a tall drug loading capacity, are biocompatible [9,10], and improve lipophilic drug absorption while also achieving effective concentration at the receptor location [11]. SLNs can bypass the apical transporter protein and enhance the therapeutic permeability [12]. They protect drugs from acidic breakdown and improve drug absorption into the vascular system [13]. The physicochemical properties of SLNs are greatly affected by several reactions such as particle size, %EE, %CDR, and loading efficiency [14].

SLNs, a possible nanotechnology-based drug delivery method, have been identified [15]. The advantages of conventional colloidal carriers should be combined with SLNs while the disadvantages are avoided. Drug targeting and controlled drug release are two highlighted benefits [16]. With an increased pharmacodynamics profile and improved drug efficacy, the carrier is biodegradable and may be used to incorporate both lipophilic and hydrophilic drugs [17].

Hadgraft et al., 2001 and Patel et al., 2012 suggested incorporating Compritol 888 ATO, a chylomicron-forming chemical, in the formulation of SLNs which enhanced glibenclamide bioavailability [18,19]. The improved bioavailability of the medication was attributed to the SLNs’ decreased efflux transport and increased surface area [18].

Dapagliflozin-loaded nanostructured lipid carriers (NLCs) were developed by Ameeduz Zafar et al., 2020 to improve oral administration. Dapagliflozin had a twofold release pattern, with a quick initial release followed by a 24 h steady-state release. In another investigation by Kazi et al., 2021, the self-nano emulsifying drug delivery systems (SNEDDS) were used to produce an oral combination dose for two anti-diabetic drugs. Dapagliflozin oral absorption in the rat model was two times more with SNEDDS than with the commercially available drug, as shown by in-vivo pharmacokinetic parameters C_max_, AUC, and oral absorption. Anti-diabetic trials demonstrated that SNEDDS significantly reduced glucose levels in diabetic mice [19].

Many researchers have reported using various statistical designs (central composite design (CCD), Box–Behnken design (BBD), D-optimal, Taguchi, 2-level factorial, Placket Burman) for formulation optimization [20,21,22,23], as it reduces the number of trials and saves time. BBD design is an innovative method for formulation optimization. The response surfaces methodology aims to understand the influence of fundamental elements on the response and attain optimal formulations that produce the desired results [24].

SLNs were explicitly created for this research to enhance dapagliflozin oral administration. SLNs were formulated using the hot homogenization approach. The dapagliflozin-loaded SLNs were produced by combining heat homogenization with ultrasonic agitation. SLNs properties such as size, distribution, surface charge, and entrapment effectiveness were analyzed. The optimized formulation will be further assessed for solid-state characterization, drug release, in vivo anti-diabetic, and biochemical evaluation.

## 2. Results

### 2.1. Design of Dapagliflozin-SLNs

#### 2.1.1. Solubility of Dapagliflozin in Lipids

**Figure 1:**
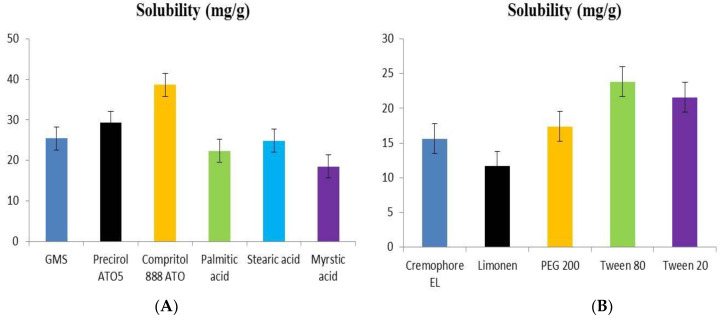
Determination of the solubility of (**A**) solid lipids, (**B**) surfactants.

The highest solubility of dapagliflozin was used to choose solid lipids for the development of SLNs. Figure 1A shows the solubility profile of dapagliflozin in various lipids. The order of dapagliflozin’s solubility in different solid lipids was observed as Compritol 888 ATO, Precirol ATO55, glyceryl monostearate, stearic acid, palmitic acid, and myristic acid. Compritol 888 ATO was preferred as the best solid lipid for manufacturing dapagliflozin-SLNs because it had the maximum partitioning of dapagliflozin [25].

#### 2.1.2. Selection of Surfactant

The screening of surfactants was done based on dapagliflozin solubility. The solubility profile of dapagliflozin in different surfactants is shown in Figure 1B. The order of solubility of dapagliflozin in surfactant is Tween 80 ≥ Tween 20 ≥ PEG200 ≥ Cremophore EL ≥ Limonene. Tween 80 was selected for the formulation of dapagliflozin-SLNs as the drug exhibited the highest solubility in Tween 80 [26].

#### 2.1.3. Selection of Sonication Time and Amplitude

Larger particles were observed at 20% amplitude compared to 40% and 50% amplitude when sonication was done for 5 min. This could be due to a lack of proper sonication time and amplitude. Whereas particle size achieved at 40% amplitude was 200 nm after 2 min of sonication time only, and a larger particle size was seen at 20% and 50% amplitude with a sonication time of 2 min, the accurate amplitude was discovered to be 40%; at an amplitude of 20%, sonication remains incomplete, and above this level (at 50%), the formulation begins to form aggregates. %EE was shown to diminish as the amplitude and sonication duration was increased steadily. As a result, the appropriate amplitude and sonication time were 40% and 2 min, respectively, which were chosen to prepare SLNs [27].

### 2.2. Preparation and Characterization of Dapagliflozin-SLNs

Dapagliflozin-SLNs were formulated using the modified high shear homogenization and ultrasonication method, after screening different concentrations of solid lipids, surfactants, and sonication time by applying hot a homogenization process with an ultrasonic phase method, as the drug exhibited enhanced solubility in the molten lipid state. It is a pretty straightforward and repeatable procedure. Seventeen batches of dapagliflozin-loaded SLNs were made using a BBD, with three independent variables: lipid content (X1), surfactant concentration (% *w*/*v*) (X2), and sonication time (X3). Table 1 shows the findings of using %EE, % CDR, and particle size as dependent variables: formulation composition of dapagliflozin SLNs using statistical BBD against independent and dependent variables [28].

#### 2.2.1. Effect of Independent Variables on %EE

**Figure 2:**
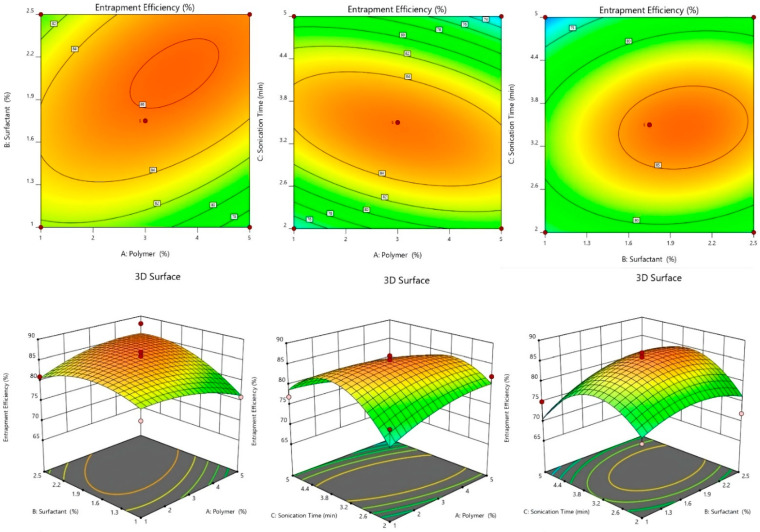
Effect of independent variables on %EE counterplots and 3D response surface plots.

The centrifugation technique was used to estimate the %EE of each experimental run of dapagliflozin-SLNs, and the findings are shown in Table 1. The impacts of variables on the %EE were shown using the polynomial equation, 3D plots, and contour plots, and the %EE was found to be in the range of 68.26 ± 0.2–94.46 ± 0.7% (Figure 2). A 2 to 5% increase in lipid concentration causes an increase in EE. The %EE increased due to the increased space for drugs in the lipid matrix. On %EE, Tween 80 showed biphasic character. Dapagliflozin increased solubility and reduced drug partition in the aqueous phase, causing an increase in %EE [29,30]. Further increase in concentration resulted in the development of micelles and an increase in dapagliflozin solubility in the aqueous phase system, and a decrease in %EE. Particle breakdown and medication leaching occur [31,32]. As a result, the %EE decreased.

EE = +86.40 − 1.25A + 2.50B − 4.25C + 0.2500AB − 8.25AC + 4.75BC − 5.82A^2^ − 0.3250B^2^ + 0.6750C^2^

The F-value of 150.25 for the model indicates that it is significant. An F-value of this magnitude has a 0.01% chance of occurring due to noise. A, B, C, AC, BC, and A^2^ are important model terms in this situation. The F-value of 50.86 for the Lack of Fit indicates that it is not significant compared to the pure error. Due to noise, a significant Lack of Fit F-value has a 10.18% chance of occurring. It is okay if there is a minor mismatch. The Adjusted R^2^ of 0.993 is reasonably close to the Predicted R^2^ of 0.916, i.e., the difference is less than 0.2. Adeq Precision measures the signal-to-noise ratio. It is preferable to have a ratio of more than four. The signal-to-noise ratio of 46.18 suggests a good signal. The design space can be navigated using this concept. The lipid concentration variable was slightly negative, indicating that as the drug concentration was increased beyond a certain point, %EE dropped, possibly due to the lipid’s inability to load a significant amount of drug. The positive coefficient of B implies that increased %EE leads to increased surfactant concentration. This could be due to the extra scope offered by acylglycerols for medicines to become entrapped. Although the lipid’s water solubility increased as the sonication time increased, %EE dropped. However, our research discovered that increasing surfactant content enhanced %EE. The growing effect is due to Tween 80, as previously discussed. It is also possible that stabilizer affects %EE [33].

#### 2.2.2. Effect of Independent Variables on %CDR

Before pharmacological testing, determining %CDR is critical for evaluating drug release from optimized SLN formulations [34]. In all formulations, the %CDR ranged from 62.83 ± 5.1 to 99.08 ± 0.4. (Table 1). For various levels of surfactant concentrations, it was seen that %CDR increased as phospholipid concentrations climbed. This variance in %CDR followed the same pattern as the change in % EE. As a result, the particle size, %EE, and %CDR of dapagliflozin-SLNs are thought to be intensely dependent on the polymer content, surfactant, and stirring speed.

%CDR = +86.40 + 0.500A − 6.75B + 5.75C + 5.75AB − 1.75AC + 6.75BC − 5.83A^2^ − 3.83B^2^ − 0.8250C^2^

At 24 h, there was an inverse link between surfactant concentration and %CDR. The decrease in drug release could be caused by an increase in lipid concentration, which causes nanoparticles to grow in size, reducing the effective surface area available to interact with the release medium. Furthermore, as the size of the nanoparticles grows, the length of the drug’s diffusion from organic to the aqueous phase grows, lowering drug release. The capacity of surfactant to reduce particle size and enhance surface area and drug release could explain an enhancement in drug release with growing lipid content [29].

The significance of the model is indicated by the Model F-value of 54.26. Model terms with *p*-values less than 0.05 are significant. B, C, AB, AC, BC, A^2^, and B^2^ are essential to model terms in this case. The model terms are not necessary if the value is bigger than 0.1. The F-value of 2.78 for the Lack of Fit indicates that it is insignificant to the pure error. Due to noise, a significant Lack of Fit F-value has a 15.64% chance of occurring. It is okay if there is a minor mismatch. The Adjusted R^2^ of 0.945 is reasonably close to the Predicted R^2^ of 0.920; the difference is less than 0.2. Adeq Precision measures the signal-to-noise ratio. A ratio of more than four is desirable. Your signal-to-noise ratio of 48.870 indicates a good signal. The design space can be navigated using this concept [30].

#### 2.2.3. Effect of Independent Variables on Particle Size

Dapagliflozin-SLNs have particle sizes that vary from 100.13 ± 7.2 to 399.08 ± 2.4 nm. The effect of a variable on particle size was discussed using the polynomial equation, 3D plots, and contour plots (Figure 2). The lipid has an agonistic effect on the size of SLNs. The size of the SLNs increased as the lipid concentration increased from 1 to 5%. The aggregation of particles causes the particles to grow in size. The second factor, Tween 80, negatively influences particle size, i.e., when the concentration is increased from 1 to 2.5%, the SLN size drops. The size reduction could be related to decrease interfacial tension between two phases, slowing particle aggregation [33]. The homogenization speed aids in particle breaking. The size of the SLNs reduced as the homogenization speed increased due to particle breakdown into smaller sizes [29].

Particle Size = +202.00 − 16.88A + 63.88B + 18.75C − 45.75AB + 40.50AC − 0.5000BC + 59.13A2 − 65.88B2 + 96.88C2

When the variable value is increased, the positive and negative coefficients of the variable in the coded equation showed an increase and decrease in the response, respectively. The model was significant, with an F-value of 33.59 and a *p*-value of 0.0001. A, B, C, BC, A^2^, B^2^, and C^2^ were significant model terms with *p* > 0.05 in the analysis of particle size (Y3). To improve the model, unimportant model terms were deleted. The adjusted R^2^ was 0.9974, while the standard R^2^ was 0.9915, reasonably close to the adjusted R^2^. Y1, with a signal-to-noise ratio of 56.9332, has adequate accuracy. The lack of fit was barely noticeable. The particle size ranged from 100.13 ± 7.2 nm to 399.08 ± 0.2.4 nm (Table 1). The equation reveals that as the lipid concentration increased, the particle size grew. The particle size was unaffected by the surfactant concentration, which was 2.5% when employed with a 5% lipid. Despite this, particle size rose considerably after reaching a concentration of >1%, which could be due to a large number of unentrapped drug particles. The positive coefficient of factor B indicated that lipids had a more significant impact on particle size. Increased lipid concentration in the SLNs may cause coalescence and increase viscosity, resulting in increased particle size [31].

Furthermore, as the lipid concentration increases, the overall surface area reduces, allowing more space for drug entrapment, resulting in larger particle size. Large particles are formed at high lipid concentrations because sonication does not perform well in more viscous liquids. On the other hand, raising the concentration of surfactant, as shown by the factor in the equation and the response surface plot, resulted in smaller particles. Because it lowers surface tension and increases surface free energy. It inhibits aggregate formation by breaking down of melting lipid droplets, resulting in smaller particles and a more stable dispersion. However, the declining effect was detected up to a certain point. A positive C^2^ result showed that increasing the sonication duration beyond the limit could cause the particle size to surpass the particle size limit by generating micelles. According to the equation, the particle size is reduced by the interaction of lipid and surfactant. This could be due to the surfactant that coats the lipid droplets and aids in reducing size. The positive coefficient of other key model factors (A^2^, C^2^) implies that a rise in surfactant, like sonication time and lipid concentration, increases particle size after a level [32].

#### 2.2.4. 3D-Response Surface and Contour Plot

**Figure 3:**
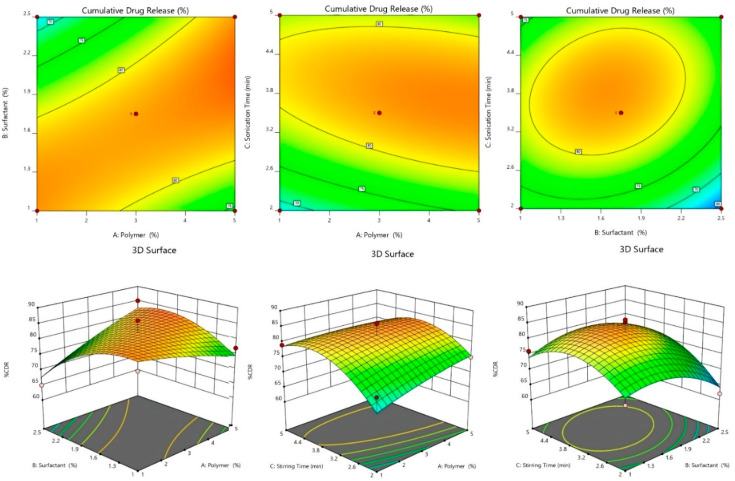
Effect of independent variables on %CDR counterplots and 3D response surface plots.

**Figure 4:**
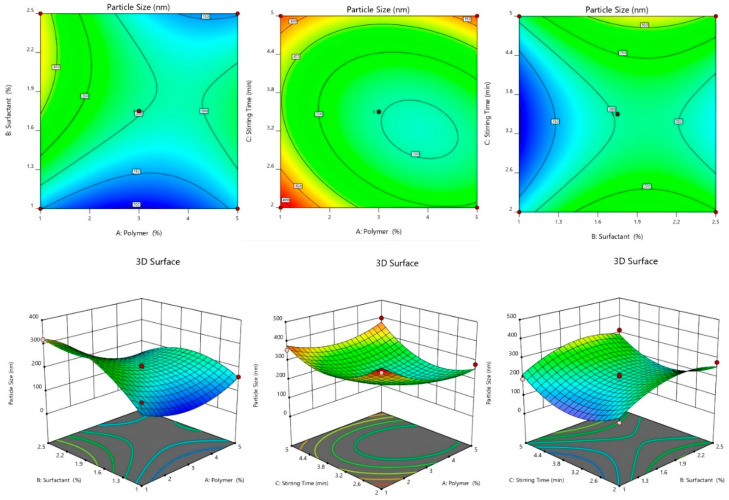
Effect of independent variables on CDR counterplots and 3D response surface plots.

As shown in Table 1, 17 experimental runs involving three factors at three levels were obtained. These plots show the effects of independent variables on a single response (Figure 2, Figure 3 and Figure 4). Table 2 shows the sum of squares, df, mean squares, F-value, *p*-value, R^2^, Lack of Fit, and residual of the best fitted quadratic model. Table 3 summarizes the statistical significance of each model (Linear, 2FI, and Quadratic) for both responses. The R^2^, Adjusted R^2^, and Predicted R^2^ values for each response model varied, with the quadratic model having the highest R^2^, so the quadratic model was chosen for each response. The optimized dapagliflozin-SLNs have a high %EE and optimal average particle size. The optimum SLNs formula was created using BBD software’s point prediction, which included lipid content (Compritol^®^ 888 ATO—1% *w*/*v*), surfactant (tween 80–20% *w*/*v*), and homogenization stirring speed (2 min). The particle size, %EE, and %CDR of optimized dapagliflozin-SLNs were determined to be 100.13 ± 7.2 nm, 94.46 ± 0.7%, and 99.08 ± 0.4%, respectively. The closeness of these expected results and actual values demonstrate the robustness of the optimization process employed for dapagliflozin-SLNs production. The optimized dapagliflozin-SLNs formulation’s PDI, zeta-potential, and drug load were 0.32 ± 0.02, −34.4 ± 1.64 mV, and 15.5 ± 0.86%, respectively.

#### 2.2.5. Optimization of SLNs Formulation

The formulation was optimized by using the BBD. The independent variables were chosen based on preliminary trial study results. Applying certain constraints, an optimized formula was generated from the software Design Expert Version 12.0.3.0. All 17 formulations with five center points and their responses are presented in Table 1. In the present study, three different responses were used in the three-factor, three-level BBD. The variables used in the study were results of optimization. The optimization results showed that the F12 formulation was 100.13 ± 7.2 nm for particle size, 94.46 ± 0.7 for %EE, and 99.08 ± 0.4 %CDR. Table 2 shows the results of an ANOVA of the fitted regression model (quadratic) for each dependent variable (Y1, Y2, and Y3). A quadratic model of all responses had a *p* < 0.0001 value, indicating that the created model was significant. The lack of fit of the model was found to be insignificant (*p* > 0.05), indicating that there is minor variation in the actual and projected values and that the model is well fitted, with independent factors having a significant effect on responses. All applicable models’ R^2^ regression values were presented, with quadratic R^2^ 0.999 being the highest (Table 3). The best-fit model for particle size, %EE, and %CDR was quadratic.

Each of the three components (lipid, surfactant, and stirring speed) is represented by the letters X1, X2, and X3 on a scale from one to three hundred. When a positive sign is used, it indicates a positive effect, and when a negative sign is used, it indicates a negative effect. When tested with 95 percent confidence, all replies revealed a statistically significant lack of match (*p* > 0.05). Other quadratic model parameters, including the remainder of the coefficients, were found to be statistically significant (*p*< 0.0001), with a high F-value and “Adeq Precision” (>4) as well. As a result, the model performed admirably when tested against acceptable data.

### 2.3. Fourier Transform Infrared (FTIR)

FTIR was used to look at the possibility of drugs interacting with the SLN components. The intensity of the peaks and the shifting of the peaks, were studied. The compatibility of the drug, Compritol ATO888, and optimized dapagliflozin-SLNs was determined using FTIR spectral analysis [34]. The characteristic bands of O–H, C=C, aromatic C–O, O–H, C=O, and C=C groups were visible in pure dapagliflozin. Pure dapagliflozin exhibited absorption peaks at 3367.10 cm^−1^ (OH stretching), 1613.16 cm^−1^ (C=C, aromatic), and 1246.70 cm^−1^ (C–O ester stretching) in the FTIR spectrum. The compounds had a 1018 cm^−1^ peak for the C–Cl bond, a 3375 cm^−1^ peak for the O–H elastic response, and a 1614 cm^−1^ peak for the C–C bond as peaks generated from dapagliflozin in common.

The absorption bands in the FTIR spectrum of the physical mixture of drug, lipid, and poloxamer 188 did not change, showing that there were no chemical interactions between the medication and excipients in the solid form, while lowering the intensity of the lipid’s carbonyl C=O group peak may indicate hydrogen bonding or dipole-dipole interaction with the medication due to electrostatic attraction. In the melting lipid, minor lipophilic dapagliflozin interaction was probably acceptable.

It was observed that the aromatic C–H and C–Cl stretchings of the drug were no longer present in the SLNs formulation (F12) as a result of the creation of hydrogen bonds, with a notable expansion of the O–H stretching at 3420 cm^−1^. When FTIR spectra of optimized dapagliflozin-SLNs were analyzed, the usual O–H stretching peak was identified at 3367.10 cm^−1^, the C=O stretching peak was found at 1246.70 cm^−1^, and the aromatic C=C stretching peak was detected at 1613.16 cm^−1^. Additionally, the C=O peak of lipid (to 1637 cm^−1^) and the aromatic C=C peak of dapagliflozin (to 1637 cm^−1^) shifted (to 1615.12 cm^−1^).

**Figure 5:**
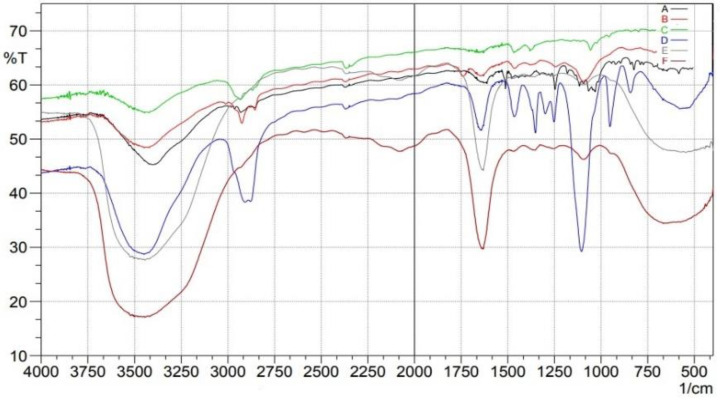
FTIR spectra studies of (**A**) pure drug, (**B**) Compritol 888ATO, (**C**) Tween 80, (**D**) Poloxamer 188, (**E**) control, (**F**) optimized formulation.

In the optimized dapagliflozin-SLNs spectra, the absolute peak of Compritol ATO888 and dapagliflozin was also present. Dapagliflozin was shown to be compatible with the components of SLNs (no interaction). Figure 5 shows the FTIR spectra of dapagliflozin (pure API), Compritol ATO888, Tween 80, and improved dapagliflozin SLNs [35]. The FTIR spectra of the optimized dapagliflozin formulation contained all of the functional group peaks found in pure drug spectra, with no extra peaks. This implies that the drug and excipients used in manufacturing the dapagliflozin-loaded SLNs had no interaction [23].

### 2.4. DSC Studies

**Figure 6:**
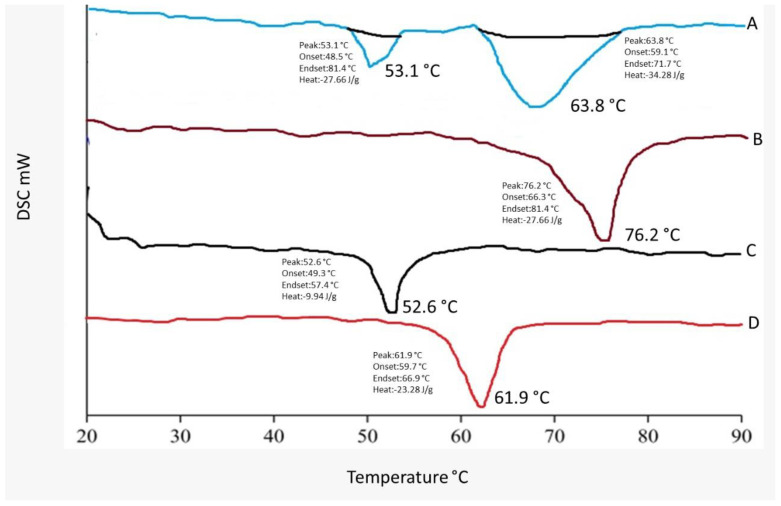
DSC thermogram of (**A**) optimized formulation, (**B**) pure drug, (**C**) Tween 80, (**D**) Compritol 888 ATO.

Differential scanning calorimetry (DSC) is a technique for measuring thermal changes in a material without any mass change. Because the exposure duration to the harsh condition was short in this experimental approach, it was difficult to detect a significant change in dapagliflozin, although the crystal form may be lost over long-term exposure, according to reference literature. Dapagliflozin’s surfactant thermogram indicated a pronounced endothermic peak at 76.2 °C and 52.6 °C (Figure 6), revealing its crystalline nature. The shift of the drug from crystalline nature to amorphous is indicated by a change in the thermal behavior of endothermic peaks of optimized formulation.

A Compritol^®^ 888 ATO melting peak at 61.9 °C was observed in improved dapagliflozin-SLNs rather than an endothermic dapagliflozin melting peak. Compritol^®^ 888 ATO endothermic peak shifted towards higher melting temperature in optimized dapagliflozin-SLNs. Compritol^®^ 888 ATO has a lower melting temperature due to nano-sized particles. It has a greater surface area than Compritol^®^ 888 ATO, lowering the melting point because melting a large crystal takes time and energy. This is due to a surfactant (tween 80) and Compritol^®^ 888 ATO’s scattered nature. Dapagliflozin did not have a melting endotherm in the thermogram of drug-loaded SLNs, indicating that the drug was entirely encapsulated in its amorphous form inside the lipid matrix of the SLNs. With the lack of the drug melting peak, the thermal profile of the dapagliflozin-loaded SLNs formulation (F12) showed a shift to 53.1 and 63.8 °C for the lipid and surfactant peaks, respectively. This revealed that the lipid’s phase transition temperature increased after being loaded with dapagliflozin. These findings revealed a strong interaction with increased amorphous drug entrapment in the SLNs. The lack of drug peaks in SLNs spectra indicates that amorphization completely encapsulated the drug. The results were consistent with those previously published [36]. As shown in Figure 6, the melting temperature, offset temperature, and the area beneath the curve is displayed on the DSC curve.

### 2.5. XRD Crystallography

**Figure 7:**
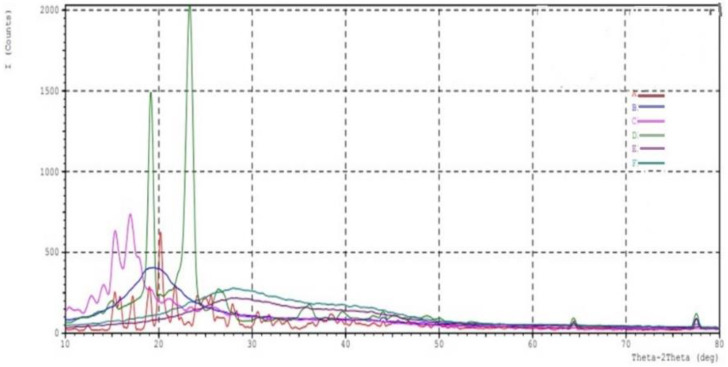
XRD pattern of (**A**) pure drug, (**B**) Compritol 888ATO, (**C**) Tween 80, (**D**) Poloxamer 188, (**E**) formulation (F4), (**F**) optimized formulation (F12).

The powder-XRD analysis confirmed the drug’s molecular dispersion state in the established formulation method. Powder-XRD was conducted to investigate the polymorphic behavior and crystallinity of dapagliflozin. Figure 7 shows the diffraction patterns of SLNs (F12) compared to Tween 80, pure dapagliflozin, and Compritol^®^ 888 ATO. The average bulk composition of the studied material after finely powdered and homogenized. Dapagliflozin XRD spectrum shows multiple intense distinct peaks at diffraction angles (2 theta degree), namely 17.2 (d-5.15), 19.0 (d-4.66), 20.2 (d-4.399), 21.9 (d-4.05), 38.0 (d-2.366), and 44.2 (d-2.04). This lattice was observed at 2° theta diffraction angles of 20.6 and 24.8.

Because of lipidic polymorphism, Compritol^®^ 888 ATO exhibited a distinctive lattice at 2° theta diffraction angles of 20.6 and 24.8. At the beginning of the diffraction patterns for both formulations, the two minor peaks belong to Compritol^®^ 888 ATO. The diffraction pattern of SLNs (F12) showed peaks intensity similar to the diffraction pattern of the blank formula. Many publications have made similar observations about the diffraction pattern of Compritol^®^ 888 ATO. Dapagliflozin’s crystalline nature was severely disturbed and was shifted to the amorphic state in optimized (F12) formulation. An X-ray powder diffraction analysis validated the results of the DSC investigation. ATO peak was identified in the XRD spectra of optimized dapagliflozin-SLNs, but no dapagliflozin peak was identified. The nano-size range of SLNs, encapsulation, and solubilization of dapagliflozin in the lipid matrix and its amorphization in the lipid matrix all contribute to this conclusion [37,38,39].

### 2.6. SEM Image Studies

**Figure 8:**
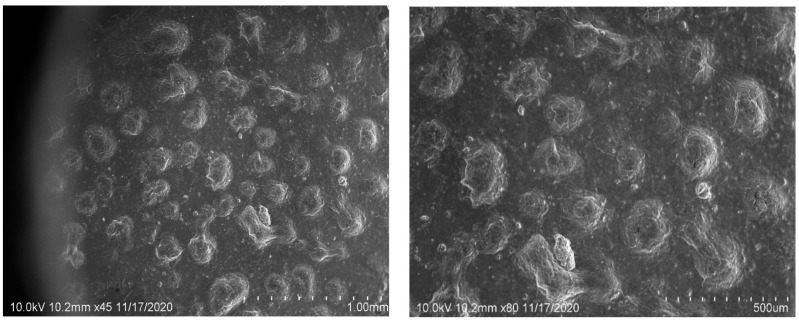
SEM micrographs of dapagliflozin-loaded SLNs of optimized formulation with different scales of measurement (10 × magnification).

SEM was used to examine the form and surface morphology of optimized dapagliflozin-SLNs, and the image revealed a spherical shape with smooth surfaces and no aggregation. As demonstrated in Figure 8, SEM describes the surface morphology of the medication and excipient [40]. This nanometric size indicates that SLNs can be absorbed by Peyer’s patches and delivered to the intestinal lymphatic system without going through the liver, increasing the drug’s oral bioavailability. Malvern’s particle size measurement is somewhat more significant than that of SEM. This can be explained in the following way: the hydrodynamic size of a nanoparticle is assessed by differential light scattering, which is the size of the nanoparticle plus the liquid layer around it, whereas the size is determined by SEM, which is the actual size of the nanoparticle. For absorption through cells and into lymphatic tissue, round and smooth particles are frequently preferred [41].

### 2.7. Zeta Potential and PDI of the Formulation

**Figure 9:**
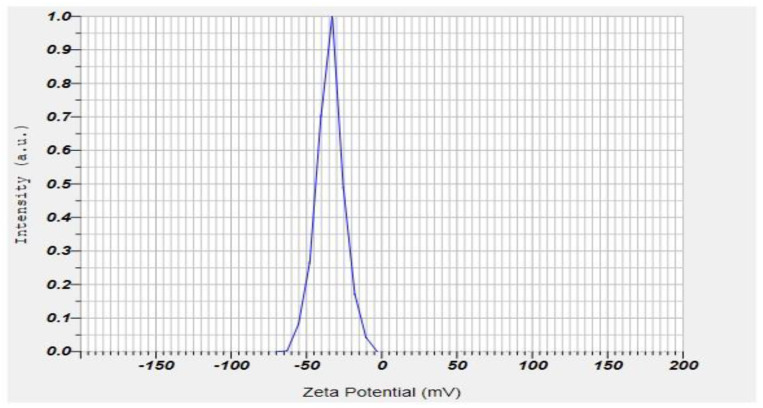
Zeta potential of dapagliflozin-loaded SLNs of optimized formulation.

The electric potential differential throughout the ionic layer around a positive ion in colloids is zeta potential. The lower the zeta potential value, the less aggregation. Their zeta potentials influence dapagliflozin-SLNs’ potential stability. The zeta potential assessment is one of the quick tests for reducing candidate formulation stability investigations, reducing experimental time and testing costs, and boosting shelf-life [42]. F1–F17 are stable if their zeta potential ranges between –22.5–34.4 ± 1.64 mV. The optimized zeta potential is −34.4 ± 1.64 mV (Figure 9).

The zeta potential indicates the electrical voltage difference in surface-charged particles and forecasts the formulation’s stability; its optimal range is more than ±30 mV. The formulation’s zeta potential (-potential) was determined in the original dispersion media. For very stable suspensions, the absolute value of -potential should be around ±30 mV. This number may be lower in the case of combination electrostatic and steric stabilization (due to the usage of ionic and non-ionic surfactants). A longer homogenization time resulted in nanoparticles with a higher zeta potential. The observed behavior can be explained by better surfactant mixing in dispersion, which results in higher zeta potential values and, as a result, improved formulation stability, as evidenced by the backscattering profiles described below [43].

For a homogenous SLNs distribution, a PDI near 0 is appropriate, and a PDI of up to 0.5 is acceptable for narrow size distribution. Our research discovered that raising the surfactant content reduced PDI (Table 2). The causes for this could be the same as for particle size. The PDI was nearly identical to the surfactant concentration, ranging from 1 to 2.5%. This means that increasing surfactant concentration decreases PDI until a certain point, which may remain unchanged. The PDI was shown to improve when the surfactant and lipid concentrations were increased. However, with the maximum quantity of lipid comprising 2.5% and 1% of the surfactant than 2 min of sonication time, the PDI was lowered, implying that the presence of free drug raises the PDI. The PDI values of 0.32 ± 0.02–0.82 ± 0.04 indicate that the system has a relatively narrow size distribution, which can be called monodisperse. The obtained results are linked with previous data presented in the literature and show that increasing the sonication time reduces the size of nanoparticles and lowers PDI values [44].

### 2.8. AFM

**Figure 10:**
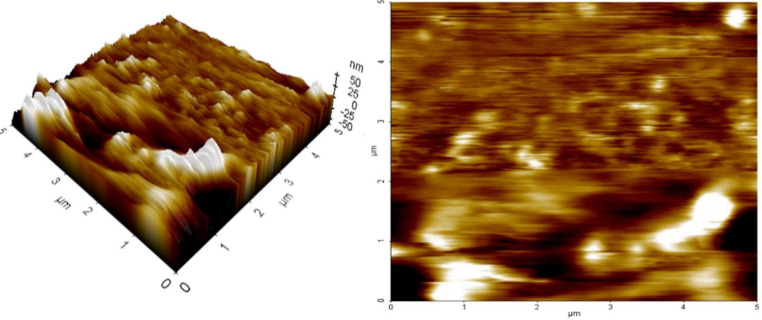
AFM analysis (within 10–15 min) on deposition on mica support.

The topology of nanocarriers was examined using AFM, which is essential when building drug delivery systems. Figure 10 shows the AFM pictures of dapagliflozin-SLNs on mica. The examination of SLNs morphology using AFM tapping and non-contact mode techniques is possible without any sample treatment such as staining, labeling, or fixation. The tip’s intermittent contact motion, in particular, reduces later or shears pressures that might otherwise deform or scrape the material. The ability to operate with greater fidelity in air or fluid in real time and on the nanometer scale is the key benefit of this approach. However, once deposited on mica support, SLNs can change shape by employing tapping mode and functioning in an aqueous solution (about 10–15 min) while still moistened and plugged in water. It depends on the vesicle composition, the contact between the sample and the substrate, and the provided sufficient tip, which might cause deformation [45].

The SLN was found to be spherical, with particles measuring roughly 200 nm in diameter, according to an AFM analysis (Figure 10). The size range reported by AFM and the size determined by dynamic light scattering is linked. The average roughness of SLN was discovered to be 10.27 nm, indicating its surface smoothness.

The outcomes of our experiments support the ideas proposed in the literature. The flattening of nanoparticles on the support was shown by comparing the diameter and height values of our SLNs just a few minutes after deposition. This revealed that the SLNs on a mica substrate were only moderately stable. Even though the diameters were more significant than the equivalent heights, the SLNs maintained a spherical, well-defined shape (Figure 10, 3D reconstruction). SLNs demonstrated a progressive tendency to change into an asymmetrical and irregular form defined as planar vesicles 20 min after deposition (data not shown). As others have discovered, this behavior can detect dried or partially dried liposomes.

### 2.9. Studies of In Vitro Drug Release

**Figure 11:**
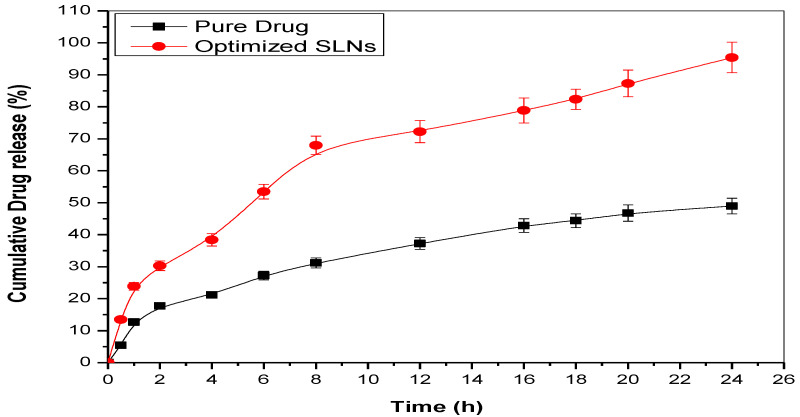
In vitro release studies on optimized SLNs and comparison with pure drug solution-Dapagliflozin.

The release pattern of optimized dapagliflozin-SLNs was compared to pure dapagliflozin under the same conditions. Optimized dapagliflozin-SLNs had a higher and long-lasting release, 69.23 ± 2.35% in 8 h, compared to a pure drug, which had a poor release pattern, 37.85 ± 4.26% (Figure 11). Dapagliflozin released from optimized dapagliflozin-SLNs had a twofold release profile, with an initial burst release within 8 h followed by a continuous release pattern [46]. The rapid release of dapagliflozin adsorbed on the surface of the SLNs resulted in this type of release behavior. Subsequently, solubilized or dispersed dapagliflozin is released slowly from the inside of the lipid core matrix via diffusion processes, resulting in a protracted drug release. The presence of Compritol^®^ 888 ATO as a solid lipid and Tween 80 as a surfactant in the SLNs resulted in much-increased drug release, which aids in the release of weakly soluble medicines. Different release kinetic models were used to suit the data. Because it has the highest R^2^, the Korsmeyer–Peppas model was chosen as the best-fit release model. It implies that the release process is controlled by diffusion, which is then followed by lipid matrix erosion [47].

### 2.10. Histopathology Studies

**Figure 12:**
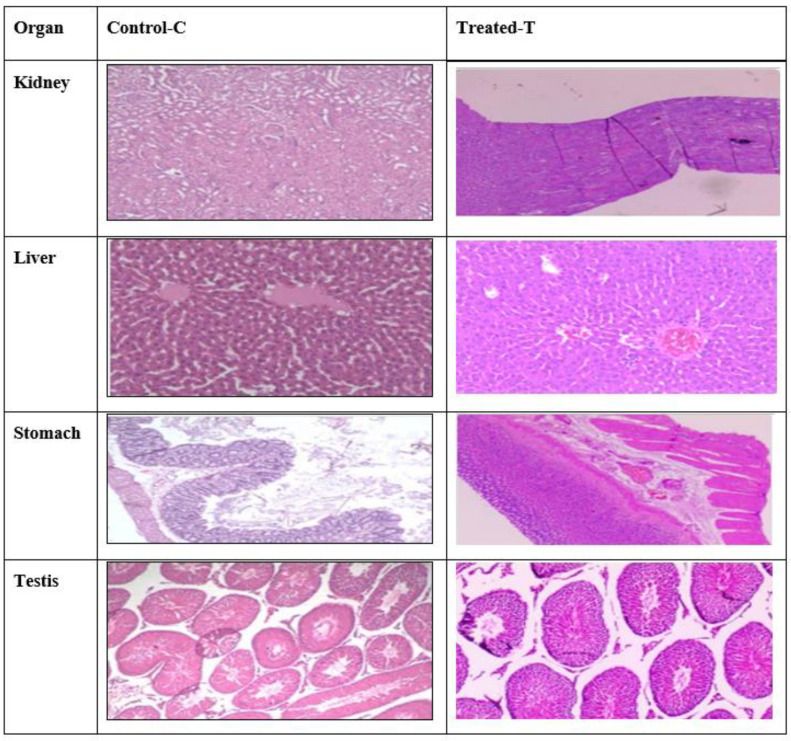
Histopathology of vital organs, namely liver, kidney, stomach, testis, and spleen in the rat during acute toxicity studies. C—Control, T—Treated. Dose, highest dose of 15 mg/kg b.wt of dapagliflozin. All sections were stained with H&E, ×400.

In acute toxicity trials, dapagliflozin-SLNs were found to be non-toxic. At any given dosage, no lethality or toxic reaction was observed. As shown in Figure 12, histopathological evidence backed up the non-toxic nature of the substance. The liver, kidneys, stomach, testis, and pancreas tissues are unaffected by dapagliflozin-SLNs.

### 2.11. In Vivo Study

Healthy male Wistar rat of either sex weighing 180 to 250 g were employed. They were kept in conventional circumstances, with a temperature of 25 °C and relative humidity of 45 to 55%.

Rats were fed with an essential pellet diet and had free access to water. All animals were carefully monitored and cared for by CPCSEA criteria for experimental animal control and monitoring.

Group I was the vehicle control, Group II was the streptozotocin (STZ) control (STZ 65 mg/kg), Groups III and IV were the testing groups, receiving 5 and 10 dosage mg/kg of dapagliflozin-SLNs, respectively, and Group V was the standard group, receiving dapagliflozin. The treatment was repeated every day for 21 days.

### 2.12. Effect of Dapagliflozin-SLNs on Insulin, HbA1c, and Blood Glucose Levels in STZ-Induced Diabetic Rats

**Figure 13:**
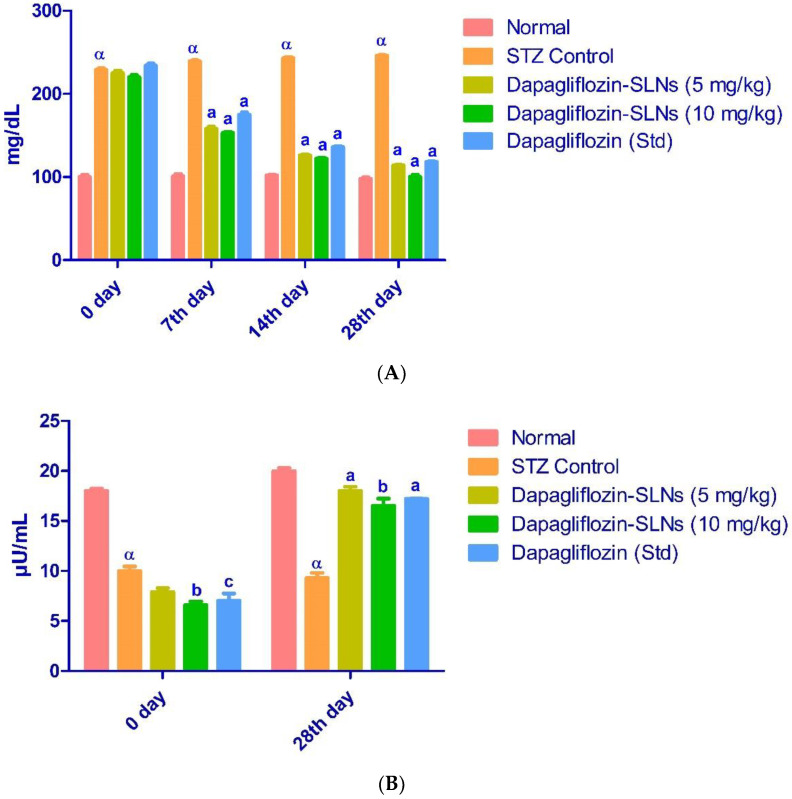
(**A**) Effect of dapagliflozin-SLNs on glucose levels in STZ-induced diabetic rats. Data are presented as the mean, standard error of the mean (*n* = 6), and analyzed using one-way ANOVA followed by Tukey’s test to compare means. *p* < 0.001 when compared to the control group; a *p* < 0.001 compared to the STZ group. (**B**) Effect of dapagliflozin-SLNs on serum insulin of STZ treated diabetic rats. Data are expressed as mean ± SEM (*n* = 6) and were analyzed by one-way analysis of variance (ANOVA) followed by Tukey’s test to compare means. α *p* < 0.001, when compared to the normal group; a *p* < 0.001, b *p* < 0.01, c *p* < 0.05 compared to STZ control.

Diabetes mellitus is characterized by high blood sugar levels that disrupt metabolism and glucose recovery over time [47,48]. STZ is commonly used to induce diabetes in laboratory animals by killing beta cells. It degrades into isocyanates and methyl diazo hydroxide before reaching the cell. Alkylation of DNA in beta-pancreatic cells by methyl diazo-hydroxide. After 28 days of treatment, blood glucose levels in groups III, IV, and V were normalized but still higher than in group I (Figure 13A,B).

**Figure 14:**
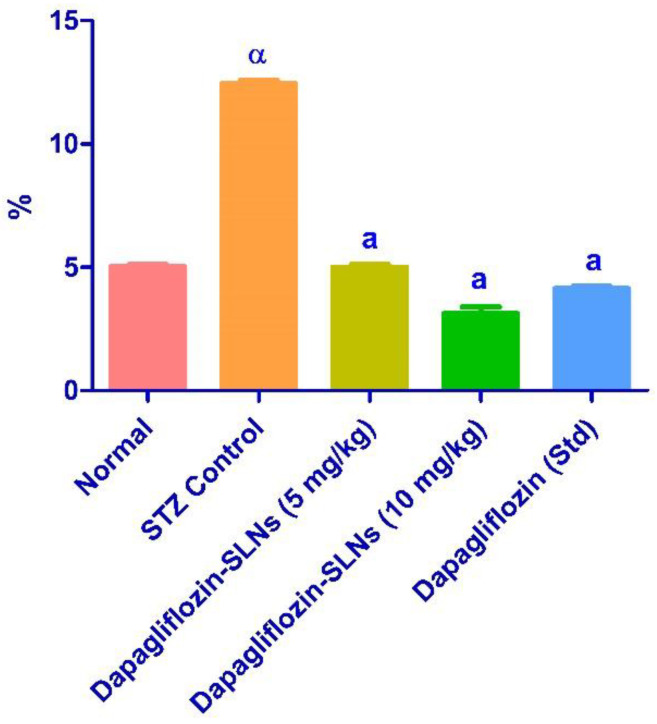
Dapagliflozin-SLNs on glycosylated hemoglobin (HbA1c) of STZ-treated diabetic rats. Data are presented as the mean, standard error of the mean (*n* = 6), and were analyzed using one-way ANOVA followed by Tukey’s test to compare means. α *p* < 0.001 when compared to the control group; a *p* < 0.001 when contrasted to the STZ group.

Insulin deficiency causes metabolic changes like higher blood glucose and better lipid profile. As shown in Figure 13B, STZ-induced diabetic rats had significantly lower insulin levels. The diabetes control group’s HbA1c was higher (*p* < 0.01) than the regular control group’s (Figure 14).

### 2.13. Lipid Profiles

**Figure 15:**
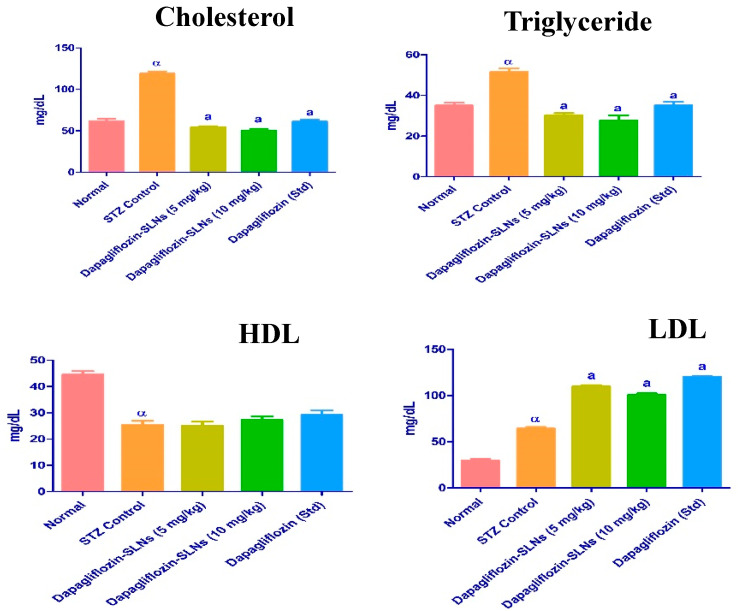
Dapagliflozin-SLNs on serum lipid profiles in STZ-induced diabetic rats. Data are presented as the mean, standard error of the mean (*n* = 6), and were analyzed using one-way ANOVA followed by Tukey’s test to compare means. α *p* < 0.001 when compared to the control group; a *p* < 0.001 compared to the STZ group.

Lipids are critical in the progression of diabetes mellitus. Hypertriglyceridemia and hypercholesterolemia are frequent lipid abnormalities in this illness. In this investigation, diabetic rats had higher plasma cholesterol, triglycerides, and LDL, which are key risk factors for cardiovascular disease [49,50]. In normal rats, STZ therapy increased total cholesterol, plasma triglycerides, LDL-C, and lowered HDL-C levels (Figure 15).

### 2.14. Biochemical Enzymes

**Figure 16:**
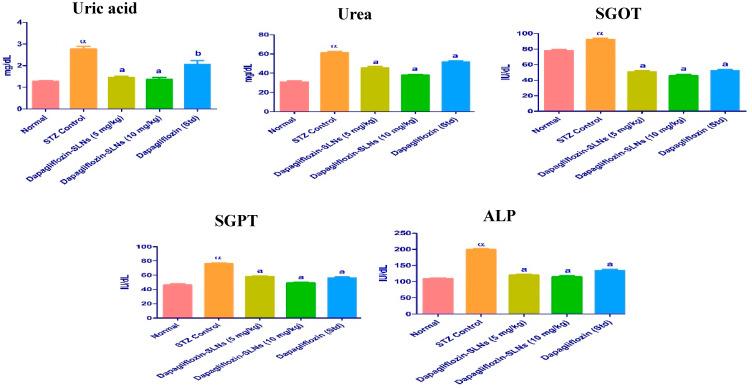
Dapagliflozin-SLNs on serum biomarkers in STZ-induced diabetic rats. Data are presented as the mean, standard error of the mean (*n* = 6), and were analyzed using one-way ANOVA followed by Tukey’s test to compare means. α *p* < 0.001 when compared to the control group; a *p* < 0.001, b *p* < 0.01 compared to the STZ group.

The decreased structural integrity of the liver produced by STZ induction and a high-fat diet has been linked to differences in SGOT, SGPT, and ALP. The liver function tests on dapagliflozin-SLNs revealed a reduction in serum ALP, SGOT, and SGPT to normal levels, demonstrating plasma membrane stability and hepatic tissue damage preparation, as shown in Figure 16.

### 2.15. Oral Bioavailability Studies

**Figure 17:**
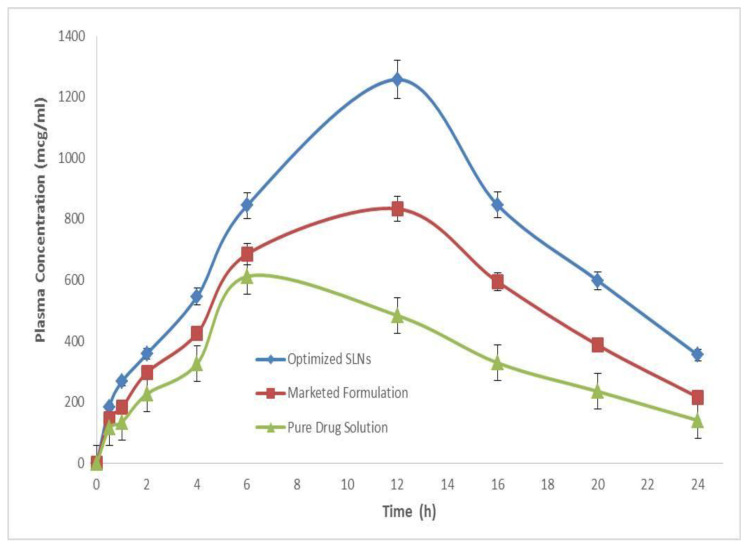
Plasma concentration vs. time profile of dapagliflozin after oral administration of optimized formulation and compared with the marketed and pure drug solution.

The in vivo pharmacokinetic behavior of the typical optimized dapagliflozin-SLNs formulation was investigated to quantify dapagliflozin in rat plasma following oral administration in the current study [51]. The study found that dapagliflozin is well absorbed following oral treatment, with peak plasma levels reaching within 8 h. Figure 17 shows that the C_max_ of dapagliflozin after oral administration of a commercial product was 621.57 ± 0.52 µg/mL. Nonetheless, the C_max_ of dapagliflozin after oral administration of our optimized dapagliflozin-SLNs was 1258.37 ± 1.21 µg/mL. The C_max_ of dapagliflozin from the SLNs formulation was enhanced significantly from 184.67 ± 3.12 to 1258.37 ± 1.21 mcg mL^−1^ (*p* < 0.05) (Figure 16). The AUC of dapagliflozin in the SLNs-treated group increased considerably from 113.03 ± 0.19 to 6310.89 ± 0.04 when compared to the sole marketed product treated group (mcg mL^−1^). Compared to the commercial development, the oral bioavailability of dapagliflozin from our optimized dapagliflozin SLNs was twofold higher in vivo testing (Table 4) [52].

These findings suggest that the proposed SLNs could improve the oral bioavailability of the anti-diabetic drug dapagliflozin, which could be used in combination with sitagliptin to treat T2DM. The results of diabetic studies show that combining dapagliflozin and sitagliptin has better efficacy and outcomes in lowering blood glucose levels. However, we only studied dapagliflozins in in vivo pharmacokinetics. For product performance, it was essential to correlate drug solubility and % age solubilized with bioavailability. This would allow for proper in vitro and in vivo drug correlation [53]. The increased bioavailability of dapagliflozin may be due to increased solubility and faster uptake of the nanoemulsion by enterocytes at the absorption site (Table 4). These findings for dapagliflozin were previously reported and are expected for sitagliptin.

### 2.16. Stability Studies

Stability is the main issue for the commercial application of SLNs. A series of stability studies (ICH guideline Q1A (R2) were carried out on the optimized formulation (F12) to determine its stability to the determining factors of vesicle size, polydispersity index, and %EE [54]. The recrystallization of the amorphous form of SLNs takes place on storage for a long time, leading to decreased drug content and release. Table 5 shows the stability results for dapagliflozin-SLNs in drug content and release.

## 3. Discussion

First, the solubility of dapagliflozin in various lipids and surfactants was investigated. The lipid Compritol 888 ATO solid lipid has the highest solubility of dapagliflozin, 38.67 ± 2.08 mg/g. Tween 80 contains the surfactant (23.84 ± 2.65 mg/g), which was chosen to produce SLNs. Tween 80 is a non-ionic surfactant that is physiologically non-toxic to humans. It is a hydrophilic surfactant with a high emulsification capability (HLB = 15).

Hot homogenization-ultra-sonication is the most straightforward and practical approach for manufacturing SLNs in laboratories. Poloxamer 188 and Compritol 888 ATO were utilized as lipids, surfactants, and co-surfactants. The homogenization time was set at 10 min at 15,000 pm, while the sonication time was 5 min at 50 W. An optimal formula was developed by applying specific constraints to the software Design Expert Version 12. The quadratic model was the best fit for all of the responses investigated, including mean particle size, %EE, and %CDR.

FTIR spectroscopy, DSC, and XRD crystallography were used to characterize the generated formulations. The pure dapagliflozin is crystalline, according to SEM images. In the improved formulation, the crystalline form of dapagliflozin was converted to an amorphous state. As illustrated in Figure 9, the size of dapagliflozin-SLNs ranges from 10 to 1000 nm.

The zeta potential is the charge that forms at the contact between a solid surface and its surrounding liquid. Stability is created when scattered particles in water have large amounts of either positive or negative zeta potential, which causes them to resist one another. Because of this, the particles become unstable due to the dispersant’s shallow zeta potential, which makes it impossible to keep them apart. F1 and F17 are stable if their zeta potential is between –22.5 to 34.4 ± 1.64 mV. The optimized zeta potential is −34.4 mv. The morphology of nanocarriers, which is crucial in designing drug delivery systems, was ascertained from the high-resolution nanoscale AFM images.

Studies on in vitro drug release, as shown in Figure 10, show that slow diffusion (release) of dapagliflozin was responsible for the first phase (burst release) and the second phase (slow release) of dapagliflozin release from the polymeric matrix.

From the results of the stability studies, it can be observed that there were no significant changes in either the release or concentration of the medicine being studied. Dapagliflozin-SLNs produced using the heat homogenization-ultrasonication process were therefore maintained in an amorphous condition throughout storage.

Dapagliflozin-SLNs were tested in STZ-induced diabetic rats in vivo to see how they affected blood glucose, insulin, and HbA1c levels. Dapagliflozin-SLNs might drop high blood glucose levels to normal in STZ-induced diabetic rats, indicating a more significant hypoglycemic effect, according to the findings of this study. In STZ and high-fat diabetic animals, dapagliflozin-SLNs reduced glucose levels. In diabetic rats, STZ significantly reduced insulin levels, as shown in Figure 12. Diabetic rats administered with dapagliflozin exhibited considerably higher insulin levels than diabetic rats in control group II. Increased insulin sensitivity could be one of the active mechanisms by which dapagliflozin-SLNs exert their anti-diabetic effects [14]

The diabetic control group’s HbA1c level was slightly higher (*p* < 0.01) than the standard control group’s HbA1c level. The dapagliflozin-SLNs and dapagliflozin significantly decreased diabetic rats’ blood levels of HbA1c compared to the control diabetic community (Figure 13). The standard glycemic regulation indicator HbA1c demonstrates the decreasing concentration in treated animals.

The lipid profile of STZ diabetic rats was considerably reduced by dapagliflozin-SLNs (*p* ≤ 0.01). Triglycerides, total cholesterol, and LDL-C levels were significantly lowered in diabetic rats administered dapagliflozin-SLNs. However, HDL-C levels in all treated diabetic rats were considerably more significant than in diabetic control groups (Figure 14). As a result, the hypolipidemic activity of dapagliflozin-SLNs has been proposed. Dapagliflozin-SLNs affected serum GOT, GPT, and ALP in normal and diabetic rats, as shown in Figure 15. According to the data, the diabetic rats had greater serum GOT, GPT, and ALP levels than the control rats. Compared to diabetic rats in the control group, dapagliflozin-SLNs administration significantly reduced serum GOT, GPT, and ALP high levels (*p* ≤ 0.01).

## 4. Materials and Methods

### 4.1. Materials

Dapagliflozin was obtained as a gift sample from Hetero Drugs Lab (Hyderabad, India). Poloxamer-188, stearic acid (SA), and cetostearyl alcohol (CSA) phospholipids were procured from Research-lab fine chem industries in Mumbai; glycerol monostearate, Mohini Organics Pvt. Ltd. (Mumbai, India), Bangalore provided Tween 80, methanol, and chloroform. SD fine chemicals Pvt. Ltd., Mumbai, India, provided Precirol^®^ ATO5 and Compritol^®^ 888 ATO.

### 4.2. Measurement of Dapagliflozin Solubility in Lipids

The solubility studies were not performed due to the solid nature of the lipids; a different method was used to determine the drug’s solubility in solid lipids. In brief, 10 mg of dapagliflozin was precisely weighed and placed in a screw-capped glass bottle with an aluminum foil covering. About 200 mg of lipid (stearic acid, cetostearyl alcohol, GMS, Precirol ATO5, and Compritol 888ATO) was added to the bottle and cooked at 80 °C while swirling continuously [8]. Then, more lipid was added in small increments while constantly stirring and heating at 80 °C until a clear solution was created. The mixture was centrifuged for 10 min at 6000 rpm (Remi centrifuge) to separate the aqueous phase. After suitable dilution in triplicate, the concentration of dapagliflozin in the aqueous phase was determined using a UV spectrophotometer (UV-1800, Shimadzu, Japan). The entire amount of lipid added to achieve a clear solution was monitored [55].

#### 4.2.1. Selection of Appropriate Surfactant

First, 1 mL surfactant (Tween 20, Tween 80, PEG200, Limonene, and Cremophore EL) was added to an Eppendorf tube, and the excess dapagliflozin and the tube were shaken for 15 min. The mixture was left to stand for 72 h in an orbital shaker. After centrifuging the mixture at 5000 rpm for 15 min, the supernatant was collected and separated [56]. After proper dilution, the amount of dapagliflozin was determined at 235 nm using a UV–Vis spectrophotometer (Shimadzu, 1800, Tokyo, Japan).

#### 4.2.2. Selection of Sonication Time and Amplitude

Probe sonication was chosen because of its better repeatability ratio and targeted intensity. By raising the ultrasonic strength and sonication time, the particle size decreases. However, if the amplitude and time increase beyond a certain point, particle size rises due to particle aggregation. The optimum amplitude and time for the sonication process were determined through experiments. 2.5% lipid, 1% surfactant, and a stabilizer were used to make SLNs. EE and particle size were measured after formulas were sonicated at 20, 40, and 50% amplitude for 2 to 5 min [57].

### 4.3. RP-HPLC Conditions for Analysis of Dapagliflozin

RP-HPLC (prominence HPLC, Shimadzu, Kyoto, Japan) with an autosampler and a UV detector (SPD 20A) set at 235 nm was used to measure dapagliflozin. The medication was separated chromatographically at room temperature using a C18 column (Phenomenex, C-18, 5 µm, 150 4.5 mm). The injection volume was set at 20 µL. Acetonitrile and water (50:50) were used as a mobile phase, with a 0.5 mL/min flow rate.

### 4.4. Preparation of SLNS

The dapagliflozin-loaded SLNs were made using a hot homogenization process with an ultrasonic phase. Dapagliflozin (100 mg) was dissolved in melted lipid (Compritol 888 ATO) and then dissolved in 20 mL chloroform: methanol (1:1) at 75 °C. The aqueous process used a surfactant called Poloxamer-188 and a co-surfactant called Tween 80, which were dissolved in 20 mL of distilled water to make a 2% solution heated to 80 °C. The heated aqueous phase solution was applied to the lipid phase held at 75 °C after the clear homogeneous lipid phase was collected in a beaker. Ultra Turrax T10 (T-10 simple ULTRA-TURRAX-IKA, Germany) was homogenized for 10 min at 15,000 rpm in a high-speed homogenizer. The pre-emulsion was subsequently ultra-sonicated at 50 w for 5 min using a probe sonicator (Frontline Sonicator). After that, the mixture was placed into cold water (between 1 and 40 °C) and mixed with a magnetic agitator. The SLNs were recrystallized at room temperature before being diluted with deionized water to make a dapagliflozin-SLN dispersion of up to 100 mL [58].

### 4.5. Experimental Design and Statistical Analysis

SLNs for dapagliflozin were optimized using a triadic, three-level BBD. Research into the quadratic solution surface and the second-order polynomial model may be carried out using this tool. Each edge of the multidimensional cube has a central point and a set of characteristics that define the exciting region. To determine the best-fitted model, all of the responses from each run were fitted to linear, 2F1, and quadratic models. The software generated contour and 3D plots, which were used to evaluate the independent factor for each answer. The actual value of each response is quantitatively compared to the software-predicted values [59]. The BBD created the generic polynomial equation for the quadratic model to check the effect of independent variables on the answer. In the synthesis of SLNs, polymer concentration (X1), surfactant concentration (X2), and stirring speed (X3) were three independent variables. Additionally, the % of drug release, EE, and particle size were selected as the dependent variables (Y1, Y2, and Y3, respectively). Runs were then conducted at various levels of each element to determine process parameters. Each run’s answers were examined using the Design EXPERT 12.0.3.0 (Table 6).

The non-linear quadratic model is given by this design as

Yi = b0 + b2X2 + b3X3 + b12X1X2 + b13X1X3 + b23X2X3 + b11X21 + b22X22 + b33X23X23

Results were analyzed using linear regression with particle size, %EE, and %CDR as response variables, lipid amount and surfactant concentration, organic phase volume, and sonication duration as factors at various levels. ANOVA and the mean effects plot for particle size and zeta potential were utilized to identify the critical variables. Pareto and contour diagrams were also used to demonstrate the effects of different factors on particle size. Design expert was used for all statistical analysis [60].

### 4.6. Measurement of Particle Size, PDI, and Zeta Potential of SLNs

Size, polydispersity index (PDI), and zeta potential (ZP) of SLNs were determined using a Zeta Sizer (Nano ZS90, Malvern, Worcestershire) (ZP). Dilution with double-distilled water was done to determine the best 50–200 kilo counts per second [61]. The calculation itself is electrophoresis of particles, with the Doppler effect of laser light dispersed by the moving particles determining the particle velocity. Using the Helmholtz–Smouches equation, the field strength was 20 V/cm; electrophoretic mobility was translated into zeta potential (mV) [62].

### 4.7. Drug Loading

Centrifugation was used to determine the drug load of the improved formulation (dapagliflozin-SLNs). The sample was deposited in a centrifuge tube and centrifuged for 30 min at 18,000 rpm with a cooling centrifuge (Sigma 3-1 KL IVD, Germany). After the supernatant was recovered, the SLNs particle was rinsed with water and dispersed in methanol. The material was then sonicated for 15 min with a probe sonicator and re-centrifuged. Finally, the supernatant was collected, and the dapagliflozin content was measured in triplicate using a UV spectrophotometer set to 235 nm. The drug loading was determined using the formula below [63].

$$\%\;Drug\;loading=\frac{Concentration\;of\;the\;drug\;in\;SLNs}{Total\;weight\;of\;SLNs}\times 100$$

### 4.8. %EE

Surfactant and co-surfactant aqueous solution-free drug concentrations were separated using a cooling centrifuge to compute the %EE. Centrifuge-controlled (Remi Instruments Ltd., Mumbai, India) decantation of the SLNs dispersion resulted in the free (unentrapped) drug sedimenting at 12,000 rpm, 4 °C for 20 min. A UV spectrophotometric method was utilized for the aqueous phase dapagliflozin concentration, and it was represented as %EE [64].

%EE = (Total Amount-Entrapped drug)/(Total amount of drug) × 100

### 4.9. Solid-State Characterizations

#### 4.9.1. FTIR

These experiments are conducted to estimate the chemical reaction between drugs and excipients. The pellet approach is used for potassium bromide (KBr), and the background spectrum is obtained in the same case. Dapagliflozin was prepared using an electrically operated KBr press model, potassium bromide (KBr) discs with the drug. Approximately 2 mg of dapagliflozin was shredded with about 5 mg of dry KBr and then pneumatically pressed into the pellet. The Fourier transform spectrometer (Shimadzu, 8400S) was used to obtain IR spectra from the prepared dapagliflozin pellet. Every spectrum is extracted from single average scans obtained against a background interferogram in the 400–4000 cm^−1^ range [65].

#### 4.9.2. DSC

DSC is conducted to estimate studies of association and polymorphism, thermotropic properties, and thermal behaviors of drugs and excipients used in the formulation. Around 5 mg of the sample was sealed and heated at 10 C/min in the aluminum pans. The temperature range of 4 to 30 °C was covered under a nitrogen atmosphere with a 100 mL/min flow rate [66].

#### 4.9.3. XRD

XRD patterns are determined for the physical mixture of the drug and other excipients to establish crystalline properties of drugs and excipients. A copper-targeted X-ray diffractometer at a voltage of 49 KV and a current of 20 MA was used to understand the crystallinity of the compound. Patterns were performed at 0.3 °C/min [67].

#### 4.9.4. SEM

The form and size of produced particles were examined using SEM. Microscopy was used to investigate dispersion patterns of nanoparticles, which were deposited on a thin carbon sheet and pumped out of the chamber. A high-intensity primary electron beam scans the sample row by row, passing through lenses that focus the electrons to a tiny point. Ionization causes secondary electrons to be generated when the concentrated electron beam reaches the location on the material. Secondary electrons are counted using a detector. A collector collects electrons positioned laterally and sends them to an amplifier [68].

#### 4.9.5. AFM

The SLNs were imaged using a Veeco NanoScope Dimension V AFM (Plainview, NY, USA) and an RT ESP Veeco tube scanner. A silicon cantilever with an ultralow-resonance resonating at 250–331 kHz and a force of 20–80 N/m was used [68]. The scanning frequency was set at 0.5 Hz. Before being seen, lipid nanoparticles were allowed to stick to a new mica surface in the solution for 24 h. After cleaning, the surfaces were washed with double-distilled water and dried in the shade [69]. A drop of dispersion sample was placed on a copper grid and dried at room temperature for one hour to conduct an examination [69].

### 4.10. In Vitro Drug Release Studies and In Vivo Anti-Diabetic Studies

The dialysis bag technique checked the release studies of optimized dapagliflozin-SLNs and pure drug solution. An enhanced open dialysis bag was used to study dapagliflozin in vitro release from improved SLNs. Before being linked to the diffusion cell, the dialysis membrane was kept for 24 h in double-distilled water at room temperature. The dialysis bag was filled with pure drug solution and optimized dapagliflozin-SLNs (equal to 2.0 mg dapagliflozin). It was submerged in 200 mL of 0.1 N HCl as a release medium for 2 h before being transferred to phosphate buffer (200 mL, pH 7.4). Throughout the experiment, the medium was continuously agitated at 60 rpm with 37 ± 0.5 °C. The aliquot (5 mL) was removed at regular intervals and replaced with newly released media in the same volume to maintain the concentration gradients. The amount of Dapagliflozin released was computed using the concentration of dapagliflozin measured using a UV spectrophotometer at each time point. The released data were fitted into multiple kinetic release models to determine the best-fit release model for improved dapagliflozin-SLNs [70].

In the in vivo investigations, SD rats of either sex (weight: 150–200 g; age: 8–10 weeks) were employed. Normal rats’ blood glucose levels were in the 80–120 mg/dL range. The rats were obtained from the National Institute of Nutrition’s animal house in Hyderabad, India. The rats were acclimated to a 12-h dark/light cycle in typical animal house facilities. Before the investigation, each animal’s blood glucose levels (BGL) were measured. For 15 days before diabetic induction, the experimental animals were provided a fat-rich meal (powdered regular pellet food, coconut oil, casein protein, vitamin, cholesterol, sucrose, sodium chloride, DL-methionine, and fructose). Their nutrition was a standard pellet meal provided by Hindustan Lever (Kolkata, India), and they had access to water at all times in clean polypropylene cages [70]. The animals were kept in a typical laboratory environment for a week before the experiment. The ethics committee approved the study protocol-with registered number-I/IAEC/NCP/013/2020-SAM.

#### 4.10.1. High-Fat Diet

With a calorie density of 13.2 kJ/g calories, the average rat diet contains 54% carbohydrates and 4% lipids. The high-fat diet had a calorie content of 22.1 kJ/g and consisted of 40% ordinary diet, 5.1% carbohydrate, 20% edible lard, 34% egg (*w*/*w*), and 0.9% salt chloride. Diabetes was produced in albino rats by feeding them a high-fat diet and administering a single modest dose (35 mg/kg) of STZ intraperitoneally. A digital glucometer was used to test the fasting BGL after 72 h of STZ treatment (Accu Check, Roche, Mannheim, Germany). Fasting BGL levels of less than 220 mg/dL were considered diabetic and were used in an experiment.

#### 4.10.2. Acute Toxicity Studies (Fixed-Dose Procedure)

Acute oral toxicity testing was performed following the 2001 OECD-420 recommendations. We used male albino Wistar rats randomly selected for the acute toxicity study. The animals were grouped into four classes (*n* = 6) and fasted overnight with free access to water. As a control, the first group received normal saline orally. Groups II, III, and IV were given the improved formulation by oral bolus at doses of 5 mg/kg, 10 mg/kg, and 15 mg/kg body weight, respectively, with filtered water. During the first 12 h after treatment, the animals were monitored at 0, 15, 30, and 60 min. They were monitored for 14 days for death and toxic symptoms. After the observation period, the surviving rats were euthanized and autopsied. The kidneys, liver, stomach, testes, and pancreas were examined.

#### 4.10.3. Induction of Diabetes

Wistar rats were placed into five classes of six after acclimatization. The first group of rats received a citrate buffer dose of 65 mg/kg (group I). Groups II, III, IV, and V diabetic rats were fed a high-fat diet for two weeks before receiving a single intraperitoneal injection of STZ (35 mg/kg b.w) (0.1 M, pH 4.5). The enhanced formulation was administered orally (with filtered water) for 28 days, beginning three days after the STZ injection. The fifth group of rats (group V) was given dapagliflozin at a dose of 1 mg/kg per day for 28 days. The rats were administered a 5% glucose solution for 48 h after STZ. Diabetic rats had blood glucose levels of 250 mg/dL or higher in subsequent testing [71].

#### 4.10.4. Biochemical Estimations

Blood samples were taken from the animals’ retro-orbital plexus after 28 days of testing. Total cholesterol, triglycerides, LDL, and HDL were determined using serum lipid profiles and blood glucose levels. ALP, SGOT, and SGPT levels were tested to identify abnormalities. A standard kit (Span Diagnostic Limited, Surat, India) was used to measure serum insulin levels, while a glycosylated hemoglobin kit was used to measure HbA1c levels (Stangen Immunodiagnostics, Hyderabad, India). At pH 7.4 and 3000 rpm/min, the livers of the rats were homogenized in a five mM Tris-HCl buffer with two mM EDTA and centrifuged at four °C for 10 min at the same temperature. MDA, CAT, GSH-Px, and SOD were analyzed using commercial kits and the manufacturers’ activity manuals in the collected supernatant [72].

#### 4.10.5. Statistical Analysis

All three results (*n* = 3) were presented as mean, standard deviation (SD). In vivo experiments used one-way ANOVA and Tukey’s tests, with results expressed as mean and standard error, mean using GraphPad Prism software. One-way ANOVA and Dunnett’s multiple comparison tests with a 95% confidence interval compared stability results to a new sample. The one-way ANOVA significance level was set at 0.05.

### 4.11. In Vivo Oral Bioavailability Studies

#### 4.11.1. Animals

The Nalgonda Pharmacy College Laboratory Animal Center provided male rats (SD, 200–220 g). A representative optimized dapagliflozin-SLNs (F12) (group A) and marketed drug (group B) were each given to six rats (group B). After a 12-h fast, the rats were given optimized dapagliflozin-SLNs (5 mg/kg). This was conducted by NIH Publications No. 80–23 (1996) and after receiving ethical clearance (No. I/IAEC/NCP/013/2020-SAM). The animals were kept in a temperature-controlled room with a 12-h light/dark cycle, with free access to food and water until 12 h before treatment.

#### 4.11.2. Pharmacokinetic Studies and Experimental Design

In the dapagliflozin investigation, the bioavailability of the enhanced SLNs was compared to that of the commercial product. At various time intervals, 0.5 mL of blood was drawn from the retro-orbital plexus in a heparinized tube (at 0.0, 0.5, 1, 2, 4, 6, 12, 16, 20, and 24 h). Blood samples were stored at 80 °C after being centrifuged for 15 min at 6000 rpm to separate the plasma. Using HPLC, a 775 L sample of plasma was mixed with a 225 L aliquot of methanol to determine the amount of dapagliflozin in plasma. After 1 min of vortex mixing, the product was centrifuged at 6000 rpm for 10 min, and the organic layer was transferred to a clean tube and dried at 45 °C under a mild nitrogen stream. Then, 225 L of mobile phase was used to re-dissolve the residue, and 5 L was injected into an HSS C18 (2.1 × 50 mm, 1.8 m) analytical column. Cmax, Tmax, and AUC (0–1) were calculated using nonlinear pharmacokinetics [73].

### 4.12. In Vitro UP-HPLC Analysis of Plasma Samples for Pharmacokinetic Assessment

The pharmacokinetic study assessed dapagliflozin concentrations in rat plasma after oral administration. Dapagliflozin, the model drug, was extracted from rat plasma using liquid-liquid extraction. Plasma was collected in 1.5 mL Eppendorf tubes. All plasma samples received 1 µg/mL internal standard solution in addition to methanol. Then, it was centrifuged for 10 min at 5000 rpm. The organic layer supernatant was transferred to a clean centrifuge tube and dried at 45–50 °C under nitrogen gas. It was reconstituted in 225 µL of the mobile phase. This was vortexed and autosampled for UPHPLC analysis. Thermo Scientific’s Di-onexVR UPHPLC system (Ultimate 3000, Bedford, MA, USA) separated the DN and plasma samples. Methanol with 0.1% formic acid and 0.2% PhA) aqueous solution (28:72) and ACN (82/18 *v*/*v*) was used as a mobile phase at 0.4 mL/min. The analysis took 6 min to complete with a 2 µL sample injection volume. Q2A I (R1), 2005) were used to validate this plasma analysis method. The method’s linearity (R^2^ = 0.9999) was validated in the 100–10,000 µg/mL range [74].

### 4.13. Stability Study

The stability study of dapagliflozin-SLNs was done as per the ICH guidelines [54]. The sample was stored in Active-vials^®^ for six months in a humidity-controlled oven (TH90S/G, Thermo lab, India) at intermediate storage conditions (30 °C 2 °C/65% RH 5% RH). The Active-vial^®^ is used to prevent moisture absorption by the samples during storage because, to avoid moisture absorption, it has a flip-top closed vial with an integrated molecular filter sleeve. The samples were withdrawn at predetermined time intervals (0, 1, 2, 3 months), and drug content and drug release were evaluated at 262 nm using a UV spectrophotometer.

## 5. Conclusions

Hot homogenization developed the SLNs followed by an ultra-sonication method using Compritol 888 ATO as lipid and Tween 80 as surfactant. The three-level, three-factor Box–Behnken experimental design was influential in optimizing dapagliflozin-SLNs. The optimized formulation showed small particle size and high %EE, optimum PDI, and zeta potential. The effect of selected independent variables on the quantity of drug release and %CDR may be predicted using the polynomial equation. Between the expected and observed values, closeness was observed. The quadratic response surface was studied for the amount of drug release, which helped understand the interaction effects of the selected independent variables and %CDR. Using the architecture of Box–Behnken to optimize the floating drug delivery system with an adequate response, a high degree of prediction was thus obtained. The IR data showed no incompatibility in the formulation between the drug and the excipients used. Optimized formulations of SLNs showed kinetics and drug release of Korsmeyer–Peppas model release. The optimized formulation was more significant (*p* < 0.05) than the pure drug solution. It confirms that the drug has higher therapeutic effectiveness after being encapsulated into SLNs.

## Figures and Tables

**Table 1:** Experimental runs conducted using BBD and values obtained for various parameters.

	Independent Variables			Dependent Variables				
F. Code	X1 (% *w*/*v*)	X2 (% *w*/*v*)	X3 (min)	Y1 (%EE)	Y2 (CDR %)	Y3 (PS) (nm)	Zeta	PDI
F1	+1	0	−1	92.06 ± 1.2	75.13 ± 2.8	280.23 ± 8.9	−27.8 ± 1.01	0.45 ± 0.05
F2	0	0	0	86.08 ± 2.3	86.06 ± 2.4	190.13 ± 4.6	−26.4 ± 0.62	0.56 ± 0.01
F3	0	−1	+1	75.31 ± 0.5	88.08 ± 0.7	189.08 ± 4.2	−22.5 ± 0.72	0.42 ± 0.06
F4	−1	−1	0	79.10 ± 0.7	89.28 ± 0.2	150.37 ± 4.6	−35.8 ± 0.22	0.37 ± 0.01
F5	+1	0	+1	68.26 ± 0.2	83.34 ± 1.1	398.49 ± 2.1	−33.4 ± 0.62	0.41 ± 0.02
F6	+1	+1	0	82.34 ± 1.8	76.29 ± 1.8	199.05 ± 2.8	−31.9 ± 1.64	0.80 ± 0.07
F7	−1	+1	0	84.61 ± 0.4	65.43 ± 2.7	320.11 ± 8.4	−38.7 ± 1.06	0.58 ± 0.01
F8	+1	−1	0	76.81 ± 2.8	77.09 ± 3.5	162.18 ± 6.2	−30.9 ± 0.62	0.41 ± 0.02
F9	0	0	0	87.12 ± 0.6	87.29 ± 4.1	210.12 ± 3.7	−33.5 ± 0.92	0.47 ± 0.01
F10	−1	0	−1	78.84 ± 1.5	73.73 ± 1.9	399.08 ± 2.4	−29.7 ± 1.08	0.80 ± 0.07
F11	0	0	0	86.09 ± 1.1	86.26 ± 3.7	202.23 ± 5.4	−28.7 ± 0.62	0.92 ± 0.10
**F12**	**0**	**−1**	**0**	**94.46 ± 0.7**	**99.08 ± 0.4**	**100.13 ± 7.2**	**−34.4 ±** **1.64**	**0.32 ± 0.02**
F13	0	0	−1	88.21 ± 0.2	84.26 ± 2.4	220.29 ± 5.1	−25.6±1.13	0.58 ± 0.01
F14	0	+1	−1	89.37 ± 1.6	62.83 ± 5.1	278.84 ± 4.9	−31.1 ± 0.72	0.82 ± 0.04
F15	0	0	−1	86.31 ± 0.5	86.13 ± 2.4	198.29 ± 3.4	−30.4 ± 1.44	0.80 ± 0.07
F16	−1	0	+1	87.94 ± 0.2	88.07 ± 1.5	355.71 ± 0.9	−32.3 ± 1.61	0.47 ± 0.01
F17	0	+1	+1	89.29 ± 1.5	87.01 ± 2.8	315.25 ± 3.4	−29.1 ± 0.62	0.41 ± 0.02

**Table 2:** Optimized quadratic model for all responses: ANOVA results.

Parameter	Source	Df	Sum of Squares	Mean of Squares	F-Values	*p*-Values
%EE	Model	9	714.83	79.43	50.86	<0.0001
	Residual	7	10.93	1.56		
	Lack of Fit	4	10.18	2.55	10.18	
	Pure Error	3	0.7500	0.2500		
%CDR	Model	9	1143.14	127.02	54.26	<0.0001
	Residual	7	16.39	2.34		
	Lack of Fit	4	15.64	3.91	15.64	
	Pure Error	3	0.7500	0.2500		
Particle size	Model	9	1.204 × 10^5^	13,377.76	33.59	<0.0001
	Residual	7	398.30			
	Lack of Fit	4	645.03	9.30		
	Pure Error	3	69.33			

**Table 3:** Regression coefficient values of the selected responses during optimization.

Y₁ (%EE)				Y₂ (CDR %)			Y₃ (PARTICLE SIZE)		
Model	R²	Adjusted R²	Predicted R²	R²	Adjusted R²	Predicted R²	R²	Adjusted R²	Predicted R²
Linear	0.296	0.134	−0.437	0.473	0.352	−0.038	0.299	0.137	−0.383
2FI	0.810	0.697	0.172	0.806	0.691	0.269	0.416	0.067	−1.646
**Quadratic**	**0.993**	**0.985**	**0.916**	**0.975**	**0.945**	**0.920**	**0.997**	**0.993**	**0.991**
Cubic	0.891	0.645		0.812	0.654		0.726	0.564	
*p*-value		<0.0001			<0.0001			<0.0001	

**Table 4:** Pharmacokinetic parameters of optimized SLNs compared with the marketed and pure drug solution.

Pharmacokinetic Parameters	Pure Drug Solution	Marketed Formulation	Optimized SLNs
Intercept	2.378	2.481	2.582
Slope	0.0019	0.0076	0.011
Co (mcg/mL)	238.850	303.024	382.68
K (h^−1^)	0.0044	0.0175	0.027
Dose (mg)	100	100	100
Dose (mcg)	100,000	100,000	100,000
Vd (mL)	41.86	33.00	26.13
Vd (L)	0.041	0.033	0.026
t1/2 (h)	155.26	39.54	25.20
Cl (L/h)	0.0001	0.0005	0.0007
AUC_o-t_ (mcg.h/mL)	59.83	54.23	95.79
AUC_1-t_ (mcg.h/mL)	8531.975	12,744.44	18,036.93
AUC_1-inf_ (mcg.h/mL)	31,425.20	12,349.3	12,885.67
AUC_total_ (mcg.h/mL)	22,833.39	449.326	5247.046
C_max_ (mcg/mL/h)	834.26	621.57	1258.37
Tmax (mL/min)	12.1	5.97	12.06

**Table 5:** Results for stability studies of dapagliflozin-SLNs.

Months	Temperature (°C)	EE (%)	CDR (%)	Drug Content (%)	Vesicle Size (nm)	Zeta Value (mv)
1st Month	Refrigeration temperature (4 ± 2 °C)	94.46 ± 0.7	99.08 ± 0.4	98.49 ± 2.1	150.37 ± 4.6	−34.4 ± 1.64
2nd Month		92.34 ± 2.1	96.45 ± 0.1	98.12 ± 1.1	167.42 ± 3.8	−32.1 ± 1.1
3rd Month		89.26 ± 0.5	92.46 ± 0.4	97.89 ± 1.3	180.64 ± 2.4	−30.9 ± 1.4
1st Month	Room temperature (30 ± 2 °C)	94.46 ± 0.7	99.08 ± 0.4	98.49 ± 2.1	150.37 ± 4.6	−34.4 ± 1.64
2nd Month		94.13 ± 0.1	99.02 ± 0.2	98.15 ± 1.1	155.26 ± 2.5	−33.1 ± 0.38
3rd Month		93.46 ± 0.4	98.19 ± 0.1	97.89 ± 0.3	160.45 ± 1.4	−32.41 ± 0.26

**Table 6:** Dependent and independent variables of SLNs.

Parameter	Units	Low (−1)	High (+1)
X1- Polymer concentration	% (*w*/*v*)	1	5
X2- Surfactant	% (*w*/*v*)	2	2.5
X3- Stirring Speed	min	2	5
Dependent Variables		Low	High
Y1- %EE		68.26 ± 0.2	94.46 ± 0.7
Y2- %CDR		62.83 ± 5.1	99.08 ± 0.4
Y3- Particle Size		100.13 ± 7.2	399.08 ± 2.4

## Data Availability

Data is contained within the article.
